# Heteroepitaxial growth of Pt and Au thin films on MgO single crystals by bias-assisted sputtering

**DOI:** 10.1038/srep23232

**Published:** 2016-03-17

**Authors:** Yulia Tolstova, Stefan T. Omelchenko, Amanda M. Shing, Harry A. Atwater

**Affiliations:** 1 Department of Applied Physics and Materials Science, California Institute of Technology, Pasadena, CA 91125, USA

## Abstract

The crystallographic orientation of a metal affects its surface energy and structure, and has profound implications for surface chemical reactions and interface engineering, which are important in areas ranging from optoelectronic device fabrication to catalysis. However, it can be very difficult and expensive to manufacture, orient, and cut single crystal metals along different crystallographic orientations, especially in the case of precious metals. One approach is to grow thin metal films epitaxially on dielectric substrates. In this work, we report on growth of Pt and Au films on MgO single crystal substrates of (100) and (110) surface orientation for use as epitaxial templates for thin film photovoltaic devices. We develop bias-assisted sputtering for deposition of oriented Pt and Au films with sub-nanometer roughness. We show that biasing the substrate decreases the substrate temperature necessary to achieve epitaxial orientation, with temperature reduction from 600 to 350 °C for Au, and from 750 to 550 °C for Pt, without use of transition metal seed layers. In addition, this temperature can be further reduced by reducing the growth rate. Biased deposition with varying substrate bias power and working pressure also enables control of the film morphology and surface roughness.

Epitaxial growth of metal films on dielectric substrates has been the subject of intense study due to its fundamental role in technologically important applications, such as optoelectronic devices and catalysis, as well as understanding crystalline growth. Material properties can vary widely with surface structure and symmetry, however oriented single crystals are expensive and often unavailable. Therefore, thin film growth is a promising tool to study orientation dependence of material properties without having to manufacture single crystalline samples. The purpose of this study was to develop oriented thin films of Au and Pt on MgO single crystalline substrates for use as ohmic heteroepitaxial templates for growth of semiconductor oxides, such as cuprous oxide, for thin photovoltaic applications. The requirements for a heteroepitaxial template include single film orientation and minimal surface roughness. Typically, metals like Au and Pt tend to adapt the (111) orientation when substrate effects are screened by surface contaminants, so high temperature processing and careful surface preparation are required to achieve epitaxy for other crystallographic orientations^1^,^2^. In this work, we grow thin films of Au and Pt in the (100) and (110) orientations with sub-nanometer surface roughness on MgO substrates while reducing the substrate temperature necessary for epitaxy using bias-assisted sputtering.

MgO is chosen as a substrate because it is transparent, refractive, insulating, and does not react with Pt or Au, forming a clean atomically flat interface. Interface mixing reactions are a common problem with metal deposition on Si, which can form silicide precipitates at the interface and impact the epitaxial relationship^3^. MgO is also well lattice matched to many metals with face-centered-cubic symmetry and can grow biaxially textured on amorphous substrates, providing a path for large-area deposition of oriented films on amorphous substrates^4^. The use of Fe or Ni layers to seed cube-on-cube epitaxy of Pt (100) on MgO (100) has been studied for catalysis applications^3^, however, these transition metals create deep level electronic defects in many semiconductors, and so are of less interest for photovoltaic film applications.

Nucleation and growth of Pt and Au particles on MgO has been subject of intense study, and reviews exist on the subject^5^. Thin film growth of Pt on MgO has been explored by molecular beam epitaxy^6^, pulsed laser deposition^3^, electron beam evaporation^7^, as well as sputtering^2^,^8^,^9^. Fewer studies of Au thin film growth on MgO exist^10^, although nucleation of Au particles has been studied extensively^11^,^12^,^13^.

We chose sputter deposition because it is an economical and industrially scalable process. In addition, sputtering allows for the use of substrate bias, which has been shown to have many beneficial effects on film growth, including *in situ* substrate cleaning and control over the energy of atoms impinging on the surface^14^. In this report, we show that substrate bias facilitates epitaxial growth of Pt and Au films on MgO by increasing mobility of surface adatoms, influencing the number and density of nucleation sites, increasing film density, and disrupting columnar grain growth. These effects result in decreasing the substrate temperature necessary to achieve epitaxial orientation.

## Methods

All of the films analyzed in this study were grown using a sputter deposition system with a base pressure of 1.3 × 10^−7^ Torr, equipped with RF and DC magnetron sputtering sources as well as an RF substrate bias capability. Single crystal MgO substrates of (100) and (110) orientation purchased commercially were annealed and cleaned *in situ* prior to deposition. The substrates were heated to 700 °C in vacuum for an hour, and then annealed in 5 mTorr of oxygen for another hour. The temperature was then ramped down at 30 °C/minute to the deposition temperature. The substrates were plasma cleaned with a RF substrate bias of 30 W (corresponding to 270 V), in 1 mTorr of 10% O_2_/90% Ar gas, for 15 minutes. At 1 mTorr, the mean free path of an Ar atom is approximately 5 cm at room temperature, and increases as the temperature increases^15^. Thus the mean free path is larger than the width of the dark space over which the plasma is non-neutral and the electric field is largest, which was measured to be approximately 3 cm. Thus Ar ions impinging on the substrate surface have energies around 270 eV. This cleaning procedure was found to result in clean MgO surfaces, as evidenced by reproducibly achieving an epitaxial relationship between the film and substrate. Pt or Au thin films were then deposited at 100 W DC power. Substrate temperature, working pressure, and RF substrate bias magnitude were varied during the study. Temperature was ramped down at a rate of 30 °C/min directly following deposition. All samples had a film thickness around 50 nm, as measured by x-ray reflectivity. Films were characterized *ex situ* using high-resolution x-ray diffractometry and atomic force microscopy.

## Results and Discussion

**Figure 1:**
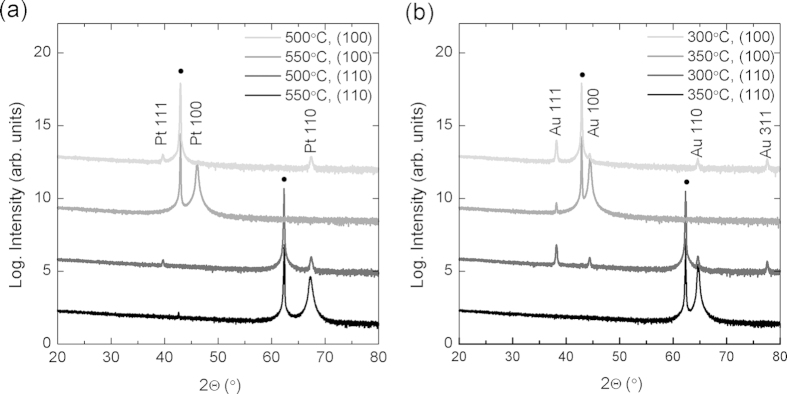
X-ray diffraction spectra of (**a**) Pt films on MgO (100) and (110), and (**b**) Au films on MgO (100) and (110). Black circles (•) indicate substrate peaks.

Figure 1 shows the orientation relationship between (a) Pt and (b) Au films grown on MgO (100) and (110) single crystals. All films in this figure were deposited with an RF substrate bias of 15 W (corresponding to 180 eV impinging atom energy). For the case of Pt films, at 500 °C both MgO orientations exhibit Pt (111), which disappears completely for 550 °C, resulting in epitaxially oriented Pt films on MgO. For the case of Au films, at 300 °C, the (111) orientation has the highest intensity on both MgO (100) and (110) substrates. At 350 °C, the dominant orientation is aligned with the substrate for both (100) and (110) MgO orientations. Although we never completely eliminate the Au (111) for growth on MgO (100), the relative magnitude of this peak suggests that the fraction of (111) orientation is less than 1%. It is important to note that without substrate bias, the temperatures necessary to achieve epitaxial orientation were found to exceed 750 °C for Pt and 600 °C for Au.

**Figure 2:**
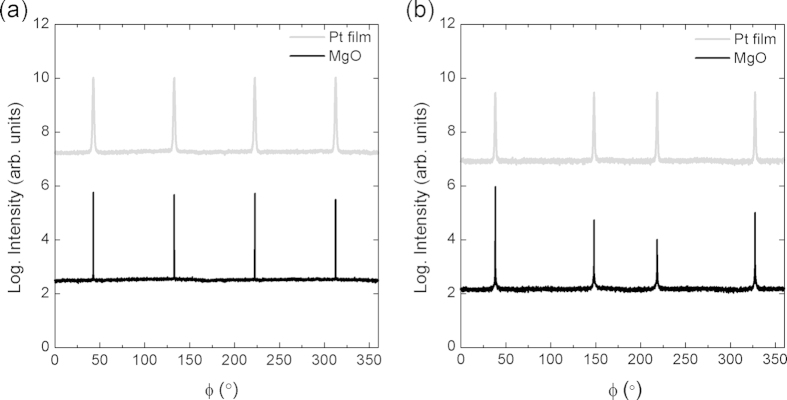
(**a**) φ scans of Pt (100) films showing Pt and MgO {111} at ψ = 54.74° showing in-plane symmetry, and (**b**) φ scans of Pt (110) films showing Pt and MgO {110} at ψ = 35.26°.

Figure 2(a) shows the φ scan of the Pt (100) film deposited at 550 °C and MgO substrate collected at a ψ tilt of 54.74° such that the {111} planes satisfy the diffraction condition. Figure 2 (b) shows the φ scan of the Pt (110) film deposited at 550 °C and MgO substrate collected at a ψ tilt of 35.26° such that the {110} planes satisfy the diffraction condition. The film is again epitaxially oriented with the substrate. The φ scans for Au (100) and (110) films show the same symmetry as that seen for Pt films. Based on these in-plane and out-of-plane orientation relationships, we conclude that the epitaxial relationship is cube-on-cube, i.e., (100)/(100) and (110)/(110).

In addition to being able to grow oriented Au and Pt films on MgO, it is essential for epitaxy that these films have minimum roughness. To provide a reference, AFM topography scans were used to measure the surface roughness of the MgO substrate before and after substrate cleaning by RF etching. Several literature studies suggest that an increase in substrate surface roughness can promote nucleation of the Pt (100) orientation on MgO (100) and suppress nucleation of the Pt (111) orientation^16^,^17^. However, in our study the root-mean-square roughness (r_RMS_) decreased after the substrate clean. For MgO (100), r_RMS_ decreased from 0.36 nm to 0.20 nm, and for MgO (110), r_RMS_ decreased from 0.17 nm to 0.13 nm. Thus we conclude that the main result of the substrate treatment, which includes heating to 700 °C, annealing in oxygen, and plasma etching the substrate, is the removal of adventitious surface contamination instead of increase of surface roughness. Without the plasma cleaning prior to deposition, we could not reproducibly achieve epitaxial orientation until heating to very high substrate temperatures and Pt and Au (111) orientation was frequently present even in these circumstances, indicating that the absence of surface contamination is essential in achieving an epitaxial growth relationship.

**Figure 3:**
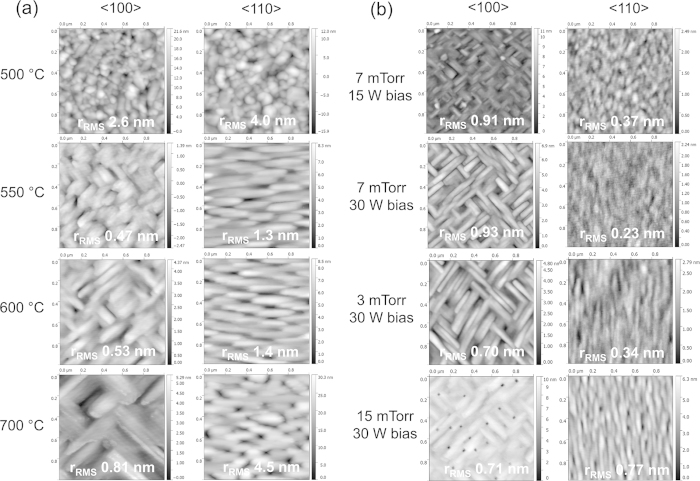
AFM topography maps of Pt films on MgO (100) and (110) deposited under different conditions. Families of directions <100> and <110> denote direction normal to the substrate surface, as well as the horizontal and vertical directions in the AFM images. (**a**) Effect of changing substrate temperature while maintaining a working pressure of 3 mTorr and bias of 15 W. (**b**) Effect of changing substrate bias and working pressure while maintaining substrate temperature at 550 °C.

Figure 3 shows the morphological features of the surface, as well as the root-mean-square roughness of Pt films deposited on different orientations of MgO. Figure 3(a) demonstrates the effect of raising the substrate temperature while maintaining a working pressure of 3 mTorr Ar, a substrate bias of 15 W, and a target power of 100 W. At 500 °C, when based on XRD analysis the film is composed of multiple crystallographic orientations, including (100), (110), and (111), the film morphology consists of three-dimensional islands and the roughness is on the order of a few nanometers. As the temperature is increased to 550 °C, and substrate-film interactions dominate the orientation relationship resulting in epitaxial growth, the morphology of the film changes drastically. In the case of the (100) MgO surface, rectangular grains with sides oriented along <110> form a crossing pattern, and the films have sub-nanometer roughness of approximately 5 Å. In the case of the (110) MgO surface, rectangular grains again grow along <110>, however no crossing of grains is evident and they are all oriented along the same direction. Film roughness is greater for this substrate orientation, at around 1.3 nm. As the substrate temperature increases, grain size increases as well, and therefore film roughness also increases. In order to attempt to decrease the roughness of Pt films grown on MgO (110), we adjusted working pressure and substrate bias while keeping the substrate temperature at 550 °C, which was the lowest temperature at which epitaxy occurs for the given growth rate. The results, illustrated in Fig. 3(b), show that increasing the substrate bias from 15 W to 30 W, or increasing the working pressure from 3 mTorr to 7 mTorr, or the combination of both, decreases surface roughness of the films into the sub-nanometer range. This can be explained by the increase in the number of heterogeneous nucleation sites due to both higher pressure, and higher energy of atom bombardment. Figure 3(b) also indicates that for growth on MgO (100) surfaces, increasing substrate bias while keeping working pressure constant increases grain size. Increased substrate bias effectively raises the energy of bombarding species and aids adatom mobility, which raises the probability of surface adatoms settling into the lowest available energy sites, thus promoting grain growth. In contrast, raising working pressure while maintaining the same substrate bias results in smaller grains due to a larger number of nucleation sites for the film and a decrease in the energy of atoms hitting the surface.

**Figure 4:**
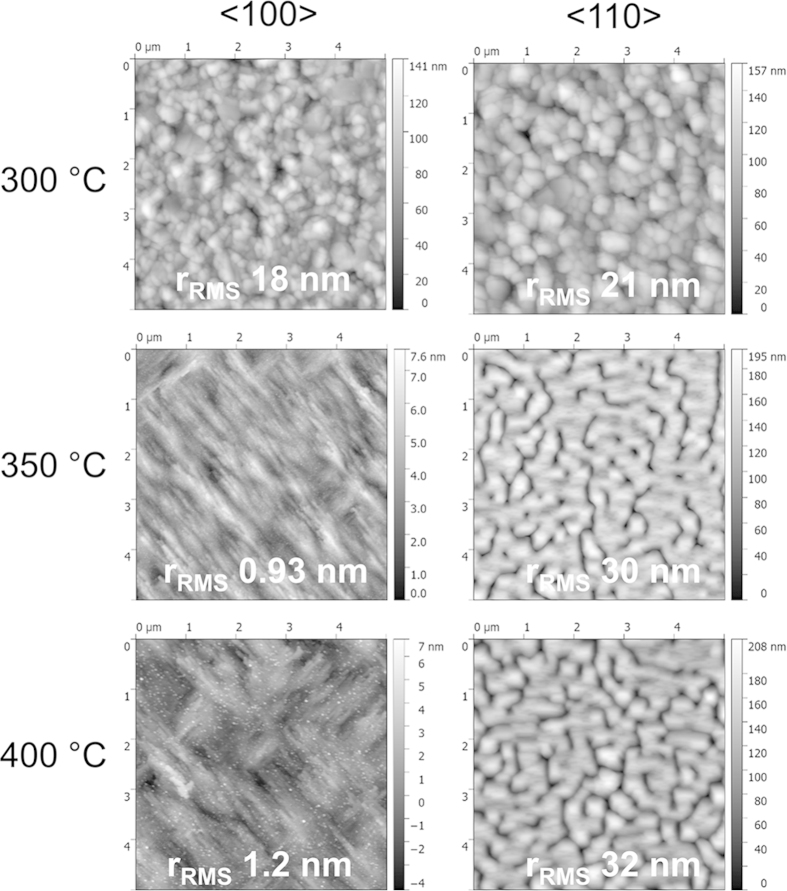
AFM topography maps of Au films on MgO (100) and (110) deposited at different substrate temperatures. All images are 5 μm by 5 μm.

Figure 4 shows the morphological features of Au films grown on MgO. At 300 °C, when the film exhibits multiple orientations, the film structure is dominated by three-dimensional islands and the roughness is about 20 nm. As the temperature is increased to 350 °C, and an epitaxial relationship is established, we can achieve Au films with sub-nanometer roughness on MgO (100) substrate. However, on the MgO (110) surface, Au islands do not form a continuous film, and therefore the roughness is on the order of film thickness. This is due to the high surface energy of the MgO/Au interface. Growing at higher working pressures and with larger bias did not result in smooth films appropriate for epitaxy.

**Figure 5:**
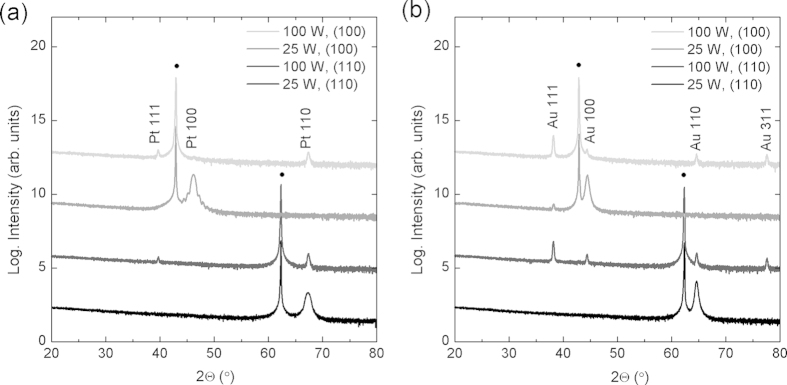
Effect of target power on the orientation relationships for (**a**) Pt on MgO grown at 500 °C and (**b**) Au on MgO grown at 300 °C. A decrease in substrate power, with a corresponding decrease in growth rate, promotes an epitaxial relationship.

Lastly, there have been literature reports showing that the substrate temperature required to achieve an epitaxial relationship decreases as the growth rate decreases^16^,^17^. Therefore, we looked at the effect of deposition rate on the temperature at which the (111) orientation disappears and the cube-on-cube orientations start to dominate. Figure 5 demonstrates the effect of deposition rate on the orientation relationships for (a) Pt on MgO grown at 500 °C and (b) Au on MgO grown at 300 °C. The growth rate is changed by changing the target power from 100 W to 25 W, keeping other deposition parameters constant. The corresponding decrease in growth rate for Pt is from 0.8 Å/s to 0.2 Å/s, and for Au is from 1.5 Å/s to 0.4 Å/s. It is indeed the case that a lower target power, and correspondingly a lower growth rate, promotes growth of epitaxial films in both Pt and Au.

## Conclusion

The main results of this work illustrate the effects of substrate preparation, temperature, and bias, on the morphology of Pt and Au thin films grown on single crystalline MgO substrates of (100) and (110) orientation. We establish an effective *in situ* substrate preparation and cleaning technique for MgO. Introduction of substrate bias during film growth provides a way to control film morphology and encourage an epitaxial growth relationship. Proper substrate preparation and bias during deposition allow us to decrease the temperature necessary to achieve oriented films from 600 to 350 °C for Au and from 750 to 550 °C for Pt. This temperature can be further decreased by decreasing the growth rate. We achieve sub-nanometer film roughness needed to make these films suitable as epitaxial templates for device fabrication. In addition, we illustrate the effects of changing the magnitude of substrate bias and working pressure on device morphology.

## Additional Information

**How to cite this article**: Tolstova, Y. *et al.* Heteroepitaxial growth of Pt and Au thin films on MgO single crystals by bias-assisted sputtering. *Sci. Rep.* **6**, 23232; doi: 10.1038/srep23232 (2016).
